# Association of anthropometric measures with kidney disease progression and mortality: a retrospective cohort study of pre-dialysis chronic kidney disease patients referred to a specialist renal service

**DOI:** 10.1186/s12882-016-0290-y

**Published:** 2016-07-08

**Authors:** Emma Davis, Katrina Campbell, Glenda Gobe, Carmel Hawley, Nicole Isbel, David W. Johnson

**Affiliations:** Centre for Kidney Disease Research, School of Medicine, University of Queensland, Translational Research Institute, Brisbane, QLD Australia; Department of Nephrology, Princess Alexandra Hospital, Brisbane, QLD Australia; Australasian Kidney Trials Network, University of Queensland, Brisbane, Australia

**Keywords:** Anthropometry, Body mass index, Chronic kidney disease, Conicity index, Mortality, Waist circumference

## Abstract

**Background:**

Although elevated body mass index (BMI) is a predictor of better clinical outcomes in dialysis patients, the evidence in pre-dialysis chronic kidney disease (CKD) is conflicting. Clinical measures of central obesity may be better prognostic indicators, although investigation has been limited. The aim of this study was to assess the predictive value of anthropometric measures for kidney failure progression and mortality in stage 3–4 CKD.

**Methods:**

The study included newly referred stage 3–4 CKD patients at a single centre between 1/1/2008 and 31/12/2010. The associations between clinical measures of obesity (BMI, waist circumference [WC] and conicity index [ConI]) and time to a composite primary outcome of doubling of serum creatinine, commencement of renal replacement therapy or mortality were evaluated using the Kaplan-Meier method and multivariable Cox regression models.

**Results:**

Over a median follow-up period of 3.3 years, 229 (25.4 %) patients of a total population of 903 experienced the composite primary renal outcome. When compared to normal BMI (18.5-24.9 kg/m^2^, *n* = 174), the risk of the composite primary outcome was significantly lower in both the overweight (BMI 25–29.9 kg/m^2^, *n* = 293; adjusted hazard ratio [HR] 0.50, 95 % CI 0.33-0.75) and obese class I/II groups (BMI 30–39.9 kg/m^2^, *n* = 288; HR 0.62, 95 % CI 0.41-0.93), but not in the obese class III group (BMI ≥40 kg/m^2^, *n* = 72; HR 0.94, 95 % CI 0.52-1.69). All-cause mortality was also lower in the overweight group (HR 0.50, 95 % CI 0.30-0.83). WC and ConI were not associated with either the composite primary outcome or mortality.

**Conclusion:**

BMI in the overweight range is associated with reduced risks of kidney disease progression and all-cause mortality in stage 3–4 CKD. WC and ConI were not independent predictors of these outcomes in this population.

**Electronic supplementary material:**

The online version of this article (doi:10.1186/s12882-016-0290-y) contains supplementary material, which is available to authorized users.

## Background

Chronic kidney disease (CKD) is a growing public health problem, with more than 10 % of the adult population in both the United States [1] and Australia [2] estimated to have stage 1–4 CKD. Targeting modifiable lifestyle factors, such as obesity, has been frequently recommended as a first line strategy for reducing the risks of kidney disease progression and cardiovascular disease (CVD) in patients with CKD [3–5]. However, whilst obesity has been identified as a risk factor for new onset kidney disease [6] and mortality in the general population [7], the evidence in CKD is conflicting. Moreover, a recent cohort study of 453,946 United States veterans with an estimated glomerular filtration rate (eGFR) < 60 ml/min per 1.73m^2^observed a consistent, U-shaped association between BMI and the outcomes of kidney disease progression and mortality, with the best outcomes observed in overweight and mildly obese subjects [8]. This risk factor paradox has been deemed by some as “reverse causation”, implying that there is unintentional illness-related weight loss which contributes to higher mortality [9].

Despite being a clinical tool which is widely used to assess obesity, BMI is an unreliable measure of body fat content in patients with CKD [10]. This measure is unable to differentiate between muscle and fat amount and distribution, and, while having reasonable correlation with body fat percentage, BMI has poor sensitivity for diagnosing obesity [11]. These limitations are particularly concerning in light of the increasing evidence that abdominal obesity is a key contributor to the health risks associated with obesity [12].

Visceral adiposity is associated with metabolic abnormalities and a pro-inflammatory state, which is linked with insulin resistance and an atherogenic lipoprotein profile [13]. Clinical measures that more precisely evaluate central obesity, such as waist circumference (WC) and conicity index (ConI) [14], have been more strongly associated with clinical outcomes than BMI in the general population [15] and in dialysis populations [16–18]. Although its prognostic value in non-dialysis CKD patients has not been well established,[19, 20] ConI, which adjusts waist circumference for height and weight, has been linked to a number of risk factors for metabolic syndrome [14] and kidney disease progression, including proteinuria and systemic inflammation [21]. It has also been linked to inflammation [22], and poor nutritional status resulting in an increased risk of mortality in haemodialysis patients [18].

The aim of this study was to investigate the associations between baseline anthropometric measures of body size (BMI, WC and ConI) and the clinical outcomes of kidney disease progression and all-cause mortality in incident Australian adults with stage 3–4 CKD referred to a specialist renal service. It was hypothesised that obesity markers that were more specific for central obesity, particularly waist circumference, would better predict progression to the composite renal end-point and death.

## Methods

The protocol for this retrospective observational cohort study was approved by Metro South Human Research Ethics Committee (HREC/10/QPAH/71) and University of Queensland School of Medicine Low Risk Ethics Committee (2014-SOMILRE-0094). General patient consent was attained at initial referral for use of clinical data in the Princess Alexandra Hospital (PAH) Nephrology Database.

### Study population

The study population consisted of patients with CKD who were referred by general practitioners or specialists to the PAH Nephrology Outpatient Department between 1 January 2008 and 31 December 2010 (catchment population approximately 1 million or 23 % of Queensland’s population). Patients included those over the age of 18 years with an eGFR of 15-59 mL/min/1.73 m^2^ whose clinical data were recorded in the PAH Nephrology Database. Patients were excluded if they had no recorded clinical or laboratory data from within three months of initial referral visit.

### Data collection

Baseline patient data from the initial visit were obtained from the PAH Nephrology Database and electronic medical records. Recorded variables included demographics, cause of kidney disease, comorbidities, medications, anthropometric measures (weight, waist circumference, height), blood pressure and laboratory values (serum creatinine, proteinuria, haemoglobin level, serum cholesterol). Height, weight and WC were measured by trained health practitioners using a standardised protocol. WC was measured at the midpoint between the lower margin of the last palpable rib and the top of the iliac crest. BMI was calculated as weight (kg) divided by height (m) squared and was categorised based on the World Health Organisation classification: underweight (<18.5 kg/m^2^), normal (18.5-24.9 kg/m^2^), overweight (25–29.9 kg/m^2^), obese class I/II (30–39.9 kg/m^2^) and obese class III (≥40 kg/m^2^). WC was divided into tertiles, stratified by gender. ConI was calculated as $\frac{WC\ (m)}{0.109\ x\ \sqrt{\frac{weight\ (kg)}{height\ (m)}}}$ and divided into tertiles.

Serum creatinine (traceable to isotopic dilution mass spectrometry) was measured by the Jaffe rate method using a Beckman DxC800 general chemistry analyser (Beckman Coulter, Brae, CA, USA). The baseline serum creatinine value from the initial referral visit was used to calculate an eGFR using the CKD-Epidemiology Collaboration (CKD-EPI) calculation [23]. Proteinuria was assessed using urine albumin-to-creatinine ratio (ACR), urine protein-to-creatinine ratio (PCR) or total protein in a 24-h urine collection and was categorised as normo, micro- or macroproteinuria according to the recommendations of the Kidney Check Australia Taskforce [24]. Urine protein was measured by immunoassay turbidimetric method and urine protein was measured by pyrogallol red and molybdate method using a Beckman DxC800 general chemistry analyser.

### Study outcomes

Patients were followed until death, loss to follow up, or 31 July 2013, whichever came first. The primary outcome of interest in the study was a composite outcome of: 1) doubling of serum creatinine; 2) initiation of renal replacement therapy (RRT); and 3) all-cause mortality. Creatinine values were followed through until 31 July, 2013 to determine if there had been an increase of more than two times the serum creatinine from initial referral, confirmed on two readings at least four weeks apart. RRT was considered as any form of dialysis or kidney transplantation. Patients who were lost to follow-up in the PAH database were cross referenced with the Australia and New Zealand Dialysis and Transplant (ANZDATA) Registry for outcome data. The ANZDATA Registry has complete capture of all patients in Australia and New Zealand who have commenced RRT since 1963 (www.anzdata.org.au).

### Statistical analysis

Results were expressed as frequencies and percentages for categorical variables, mean ± standard deviation for continuous normally distributed variables, and median [interquartile range; IQR] for continuous variables that were not normally distributed. Categorical data were compared using chi-square tests. Continuous normally distributed data were compared using two tailed unpaired t-tests. Continuous non-normally distributed data were compared using Mann–Whitney tests. The association between anthropometric indices and time to either the composite primary end-point or mortality were evaluated by Kaplan Meier and multivariable Cox proportional hazards model analyses. Each anthropometric index was analysed as a categorical variable in the primary analysis and as a continuous variable in a sensitivity analysis. For the multivariable analyses, several models were examined: Model 1, adjusted for age (continuous); Model 2, adjusted for age, gender and race (Caucasian vs. non-Caucasian); and Model 3 included age, gender, race, cause of CKD (categorised based on the Study of Heart and Renal Protection [SHARP] trial [25]: diabetic nephropathy, glomerulonephritis, cystic kidney disease and other), eGFR, proteinuria, and presence of diabetes as a comorbidity. The data was further analysed with stratification by gender. Patients with missing data were excluded from multivariable analysis. *P*-values of <0.05 were considered significant. Statistical analyses were performed using SPSS Statistics version 21.

## Results

### Baseline characteristics

**Fig. 1:**
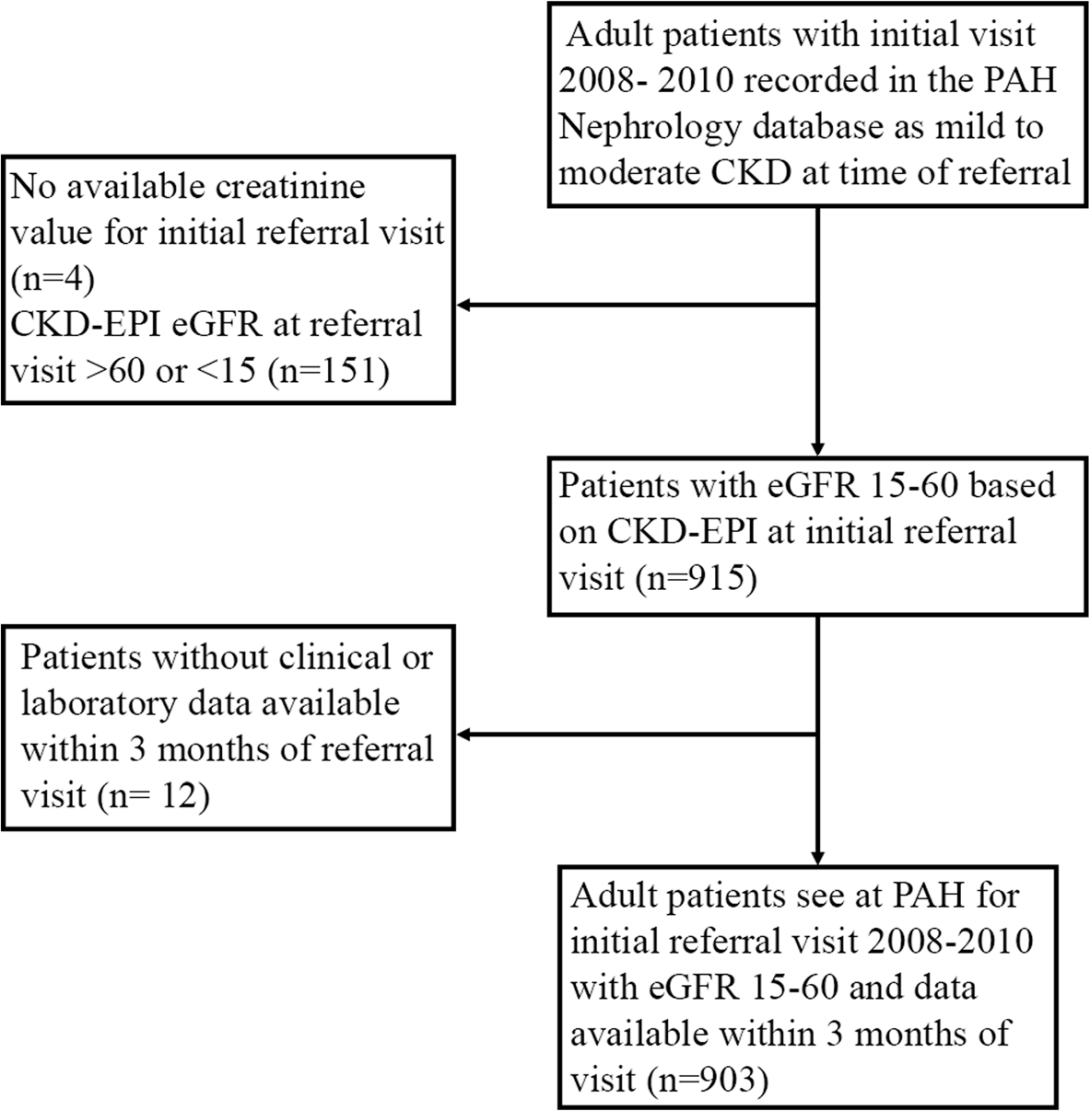
Derivation of the Study Cohort. PAH, Princess Alexandra Hospital; CKD, chronic kidney disease; CKD-EPI, CKD-Epidemiology Collaboration [30]; eGFR, estimated glomerular filtration rate

Of 1070 adult patients with CKD referred to the centre between 2008 and 2010, a total of 903 patients were included in the study (Fig. 1). Their baseline characteristics are shown in Table 1.

**Table 1:** Baseline characteristics of the stage 3–4 CKD study cohort based on BMI category

Variable	Total population	Breakdown by BMI (kg/m^2^)					
		<18.5	18.5-24.9	25-29.9	30-39.9	≥40	*p*-value*
	(*n* = 903)	(*n* = 15; 1.8 %)	(*n* = 174; 20.7 %)	(*n* = 293; 34.8 %)	(*n* = 288; 34.2 %)	(*n* = 72; 8.6 %)	
Male gender	515 (57 %)	2 (13.3 %)	96 (55.2 %)	181 (61.8 %)	169 (58.7 %)	35 (48.6 %)	0.002
Age (years)	66.26 ± 13.62	56.5 ± 18.8	67 ± 15.7	68.3 ± 13	65.4 ± 12.3	61.0 ± 11.1	<0.001
Race							0.036
Caucasian	708 (78.4 %)	11 (73.3 %)	137 (78.7 %)	241 (82.3 %)	226 (78.5 %)	51 (70.8 %)	
Non-Caucasian	115 (12.7 %)	2 (13.3 %)	29 (16.6 %)	24 (8.2 %)	36 (12.5 %)	14 (19.4 %)	
Not stated	80 (8.9 %)	2 (13.3 %)	8 (4.6 %)	28 (9.6 %)	26 (9.0 %)	7 (9.7 %)	
Cause of CKD							<0.001
Diabetic nephropathy	243 (26.9 %)	0 (0.0 %)	32 (18.4 %)	59 (20.1 %)	98 (34.0 %)	33 (45.8 %)	
Glomerulonephritis	75 (8.3 %)	2 (13.3 %)	17 (9.8 %)	23 (7.8 %)	20 (6.9 %)	5 (6.9 %)	
Cystic kidney disease	26 (2.9 %)	1 (6.7 %)	7 (4.0 %)	13 (4.4 %)	4 (1.4 %)	1 (1.4 %)	
Other	559 (61.9 %)	12 (80.0 %)	118 (67.8 %)	198 (67.6 %)	166 (57.6 %)	33 (45.8 %)	
Comorbidities							
Diabetes mellitus (*n* = 902)	387 (42.9 %)	0 (0.0 %)	49 (28.2 %)	114 (28.9 %)	146 (50.7 %)	47 (66.2 %)	<0.001
CAD (*n* = 823)	270 (32.8 %)	1 (7.1 %)	49 (31.0 %)	93 (33.6 %)	83 (32.5 %)	21 (33.9 %)	0.351
CLD (*n* = 872)	117 (13.4 %)	1 (6.7 %)	21 (12.4 %)	38 (13.5 %)	36 (13.1 %)	16 (22.5 %)	0.228
CBVD (*n* = 870)	110 (12.6 %)	0 (0.0 %)	17 (10.2 %)	39 (13.9 %)	40 (14.2 %)	8 (11.6 %)	0.393
PVD (*n* = 854)	140 (16.4 %)	0 (0.0 %)	20 (12.2 %)	46 (16.5 %)	54 (19.8 %)	12 (17.9 %)	0.115
Medication use							
Lipid lowering (*n* = 839)	402 (47.9 %)	0 (0.0 %)	72 (43.4 %)	127 (46.4 %)	143 (54.2 %)	38 (56.7 %)	<0.001
ACEi/ARB (*n* = 839)	509 (60.7 %)	6 (40.0 %)	93 (56.0 %)	166 (60.6 %)	168 (63.6)	50 (74.6 %)	0.032
Antihypertensive (*n* = 822)	655 (79.7 %)	8 (53.3 %)	125 (75.8 %)	220 (80.9 %)	217 (82.2 %)	56 (84.4 %)	0.033
EPO (*n* = 839)	29 (3.5 %)	1 (6.7 %)	8 (4.8 %)	10 (3.6 %)	8 (3.0 %)	2 (3.0 %)	0.863
eGFR (*n* = 903)	37.9 ± 11.7	38.3 ± 12.2	38.3 ± 12.4	37.4 ± 11.3	37.9 ± 11.2	39.9 ± 12.0	0.573
Stage 3	654 (72.4 %)	11 (1.8 %)	124 (20.2 %)	210 (34.3 %)	212 (34.6 %)	56 (9.1 %)	
Stage 4	249 (27.6 %)	4 (1.7 %)	50 (21.8 %)	83 (36.2 %)	76 (33.2 %)	16 (7.0 %)	
Proteinuria (*n* = 780)							0.493
Microproteinuria	459 (58.8 %)	6 (54.5 %)	88 (59.5 %)	163 (64.2 %)	138 (54.3 %)	40 (59.7 %)	
Macroproteinuria	293 (37.6 %)	5 (45.5 %)	56 (37.8 %)	83 (32.7 %)	104 (40.9 %)	23 (34.3 %)	
Obesity measures							
WC (*n* = 594)	101.6 ± 16.1	69.4 ± 6.9	86.1 ± 8.2	98.5 ± 8.2	111.2 ± 10.3	130.6 ± 12.6	<0.001
ConI (*n* = 588)	1.33 ± 0.10	1.21 ± 0.11	1.28 ± 0.09	1.33 ± 0.09	1.36 ± 0.09	1.39 ± 0.09	<0.001

Results expressed as mean ± SD or number (percentage). The number of patients with data available follows the measured variable, if total population data not available

*CKD* chronic kidney disease, *BMI* body mass index, *CAD* coronary artery disease, *CLD* chronic lung disease, *CBVD* cerebrovascular disease, *PVD* peripheral vascular disease, *ACEi* angiotensin-converting-enzyme inhibitor, *ARB* angiotensin II receptor blocker, *EPO* erythropoietin, *eGFR* estimated glomerular filtration rate, *WC* waist circumference, *ConI* conicity index

*Differences between BMI categories were assessed by chi-squared test or ANOVA, depending on the data type

### Primary composite end-point

During a median follow-up of 3.3 years, 229 patients (25.4 %) reached a composite primary outcome, including 15.7 % who died, 8.3 % who experienced a doubling of their serum creatinine and 1.3 % who commenced RRT as the initial event. The median time to the primary end-point was 1.81 years.

**Fig. 2:**
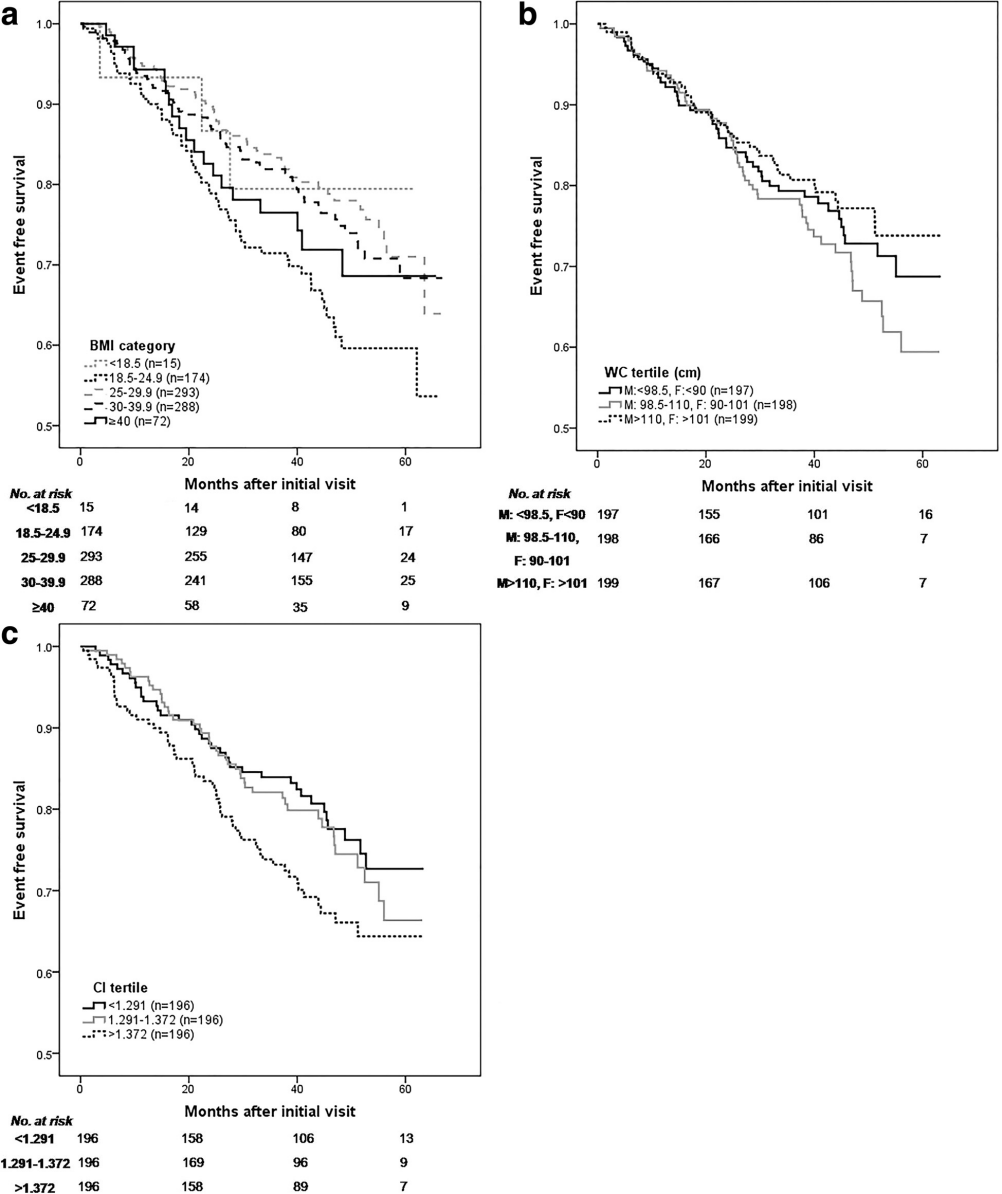
Kaplan-Meier Curves for Progression to the Primary Outcome for Clinical Anthropometric Measures. The primary outcome included, doubling of serum creatinine, commencement of renal replacement therapy or all-cause mortality, with anthropometric measures of body mass index (BMI), waist circumference (WC) and conicity index (ConI). Shown below the graphs are the number of patients at risk. **a**. BMI by categories (kg/m^2^) [Log rank score 10.44, *p* = 0.034]. **b**. WC by tertiles [Log rank score 3.09, *p* = 0.21]. **c**. ConI by tertiles [Log rank score 4.13, *p* = 0.13]

**Fig. 3:**
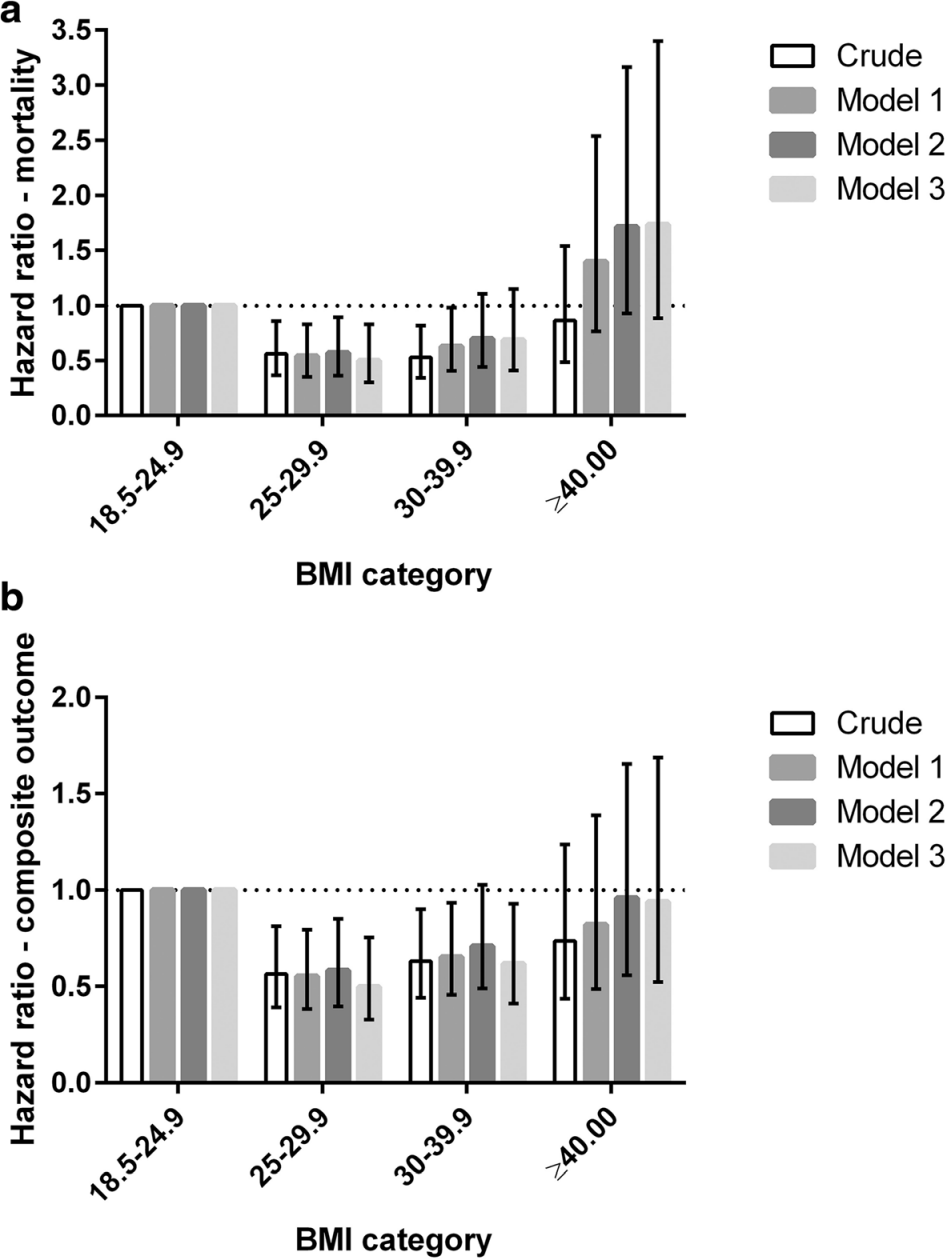
Hazard Ratios for the Composite Outcome (**a**) and Mortality (**b**) for BMI Categories. A U-shaped association is evident between BMI categories and hazard ratios for both the composite outcome and mortality, in stage 3–4 CKD patients. Hazard ratios with 95 % CIs shown for Cox regression models: crude, model 1 (age-adjusted), model 2 (model 1 plus gender and race-adjusted), model 3 (model 2 plus estimated glomerular filtration rate, proteinuria, cause of chronic kidney disease, diabetes status)

On univariable analysis, patients with a BMI in the overweight range (hazard ratio [HR] 0.565, 95 % CI 0.39-0.81, *p* < 0.01) and in the class I/II obese range (HR 0.63, 95 % CI 0.44-0.90) experienced a lower hazard of the primary end-point compared with normal weight individuals (Fig. 2, Table 2). Similar results were observed following multivariable Cox proportional hazards model analysis (Table 2, Fig. 3).

**Table 2:** Association between baseline obesity parameters and the primary outcome in stage 3–4 chronic kidney disease. Comparison performed using Cox proportional hazards modelling to compare body mass index, waist circumference and conicity index with the composite outcome of doubling of serum creatinine, commencement of renal replacement therapy or all-cause mortality

		HR (95 % CI)			
	No. (%)	Crude	Model 1	Model 2	Model 3
Body mass index (kg/m^2^)					
<18.5	15 (1.8)	0.54 (0.17-1.71)	0.43 (0.2-1.99)	0.57 (0.14-2.38)	0.74 (0.17-3.19)
18.5-24.9	174 (20.7)	1.00 (referent)	1.00 (referent)	1.00 (referent)	1.00 (referent)
25-29.9	293 (34.8)	0.57 (0.39-0.81)**	0.55 (0.38-0.79)**	0.58 (0.40-0.85)**	0.50 (0.33-0.75)**
30-39.9	288 (34.2)	0.63 (0.44-0.90)*	0.65 (0.46-0.93)*	0.71 (0.49-1.03)	0.62 (0.41-0.93)*
≥40	72 (8.6)	0.74 (0.44-1.24)	0.82 (0.49-1.39)	0.96 (0.56-1.65)	0.94 (0.52-1.69)
Waist circumference tertiles (cm)					
M: <98.5, F < 90	197 (33.2)	1.00 (referent)	1.00 (referent)	1.00 (referent)	1.00 (referent)
M: 98.5-110, F: 90-101	198 (33.3)	1.23 (0.83-1.82)	1.14 (0.75-1.65)	1.31 (0.86-1.97)	0.99 (0.63-1.56)
M > 110, F: >101	199 (33.5)	0.87 (0.57-1.33)	0.89 (0.58-1.35)	1.00 (0.64-1.57)	0.87 (0.54-1.41)
Conicity index tertiles					
<1.291	196 (33.3)	1.00 (referent)	1.00 (referent)	1.00 (referent)	1.00 (referent)
1.291-1.372	196 (33.3)	1.14 (0.74-1.80)	1.03 (0.67-1.59)	1.03 (0.65-1.63)	1.13 (0.68-1.86)
>1.372	196 (33.3)	1.59 (1.05-2.39)*	1.46 (0.96-2.20)	1.39 (0.88-2.22)	1.16 (0.69 -1.95)

Results expressed as number (percentage) and hazard ratio (95 % confidence interval)

Model 1: Adjusted for age

Model 2: Adjusted for age, gender, race (Caucasian vs. Non-Caucasian)

Model 3: Model 2 + estimated glomerular filtration rate, proteinuria, cause of chronic kidney disease, diabetes status

*P ≤ 0.05, **P ≤ 0.01

WC did not show a significant association with the primary outcome as either a continuous or a categorical variable in univariable analysis (Fig. 2, Table 2). Multivariable models also failed to identify a significant relationship between WC and the primary end-point.

In univariable survival analysis, increasing ConI was predictive of a higher risk of the composite outcome (Fig. 2, Table 2). Using ConI as a continuous variable, each 0.1 unit increase in ConI was associated with a 28 % increased risk of progression to the composite outcome (HR, 1.278; 95 % CI, 1.07-1.53; *P* < 0.01).

### Mortality

The relationship between different anthropometric measures and all-cause mortality are shown in Table 3. Those patients in the overweight BMI category had a lower risk of mortality. There was evidence of a U-shaped association, with BMI ≥40 kg/m^2^ showing increasing risk of mortality (Fig. 3).

**Table 3:** Association between baseline obesity parameters and all-cause mortality in stage 3–4 chronic kidney disease. Comparison performed using Cox proportional hazards modelling to compare body mass index, waist circumference and conicity index with all-cause mortality

		HR (95 % CI)			
	No. (%)	Crude	Model 1	Model 2	Model 3
Body mass index (kg/m^2^)					
<18.5	15 (1.8)	0.23 (0.03-1.67)	0.39 (0.05-2.89)	0.49 (0.07-3.58)	1.03 (0.14-7.80)
18.5-24.9	174 (20.7)	1.00 (referent)	1.00 (referent)	1.00 (referent)	1.00 (referent)
25-29.9	293 (34.8)	0.56 (0.37-0.86)**	.54 (0.35-0.83)**	0.57 (0.36-0.90)*	0.50 (0.30-0.83)**
30-39.9	288 (34.2)	0.53 (0.34-0.82)**	.63 (0.41-0.98)*	0.70 (0.44-1.11)	0.69 (0.41-1.15)
≥40	72 (8.6)	0.86 (0.49-1.54)	1.40 (0.77-2.54)	1.71 (0.93-3.16)	1.74 (0.89-3.40)
Waist circumference tertiles (cm)					
M: <98.5, F < 90	197 (33.2)	1.00 (referent)	1.00 (referent)	1.00 (referent)	1.00 (referent)
M: 98.5-110, F: 90-101	198 (33.3)	1.21 (0.75-1.95)	1.04 (0.65-1.68)	1.18 (0.72-1.95)	0.90 (0.52-1.58)
M > 110, F: >101	199 (33.5)	0.83 (0.50-1.40)	1.07 (0.59-1.71)	1.09 (0.62-1.92)	1.00 (0.54-1.86)
Conicity index tertiles					
<1.291	196 (33.3)	1.00 (referent)	1.00 (referent)	1.00 (referent)	1.00 (referent)
1.291-1.372	196 (33.3)	1.61 (0.93-2.77)	1.29 (0.75-2.22)	1.33 (0.74-2.36)	1.67 (0.89-3.13)
>1.372	196 (33.3)	1.85 (1.09-3.15)*	1.57 (0.92-2.67)	1.53 (0.84-2.79)	1.31 (0.66-2.58)

Results expressed as number (percentage) and hazard ratio (95 % confidence interval)

Model 1: Adjusted for age

Model 2: Adjusted for age, gender, race (Caucasian vs. Non-Caucasian)

Model 3: Model 2 + estimated glomerular filtration rate, proteinuria, cause of chronic kidney disease, diabetes status

*P ≤ 0.05, **P ≤ 0.01

There was no direct relationship between WC and all-cause mortality in either univariable or multivariable analysis.

ConI was also predictive of mortality (HR 1.413, 95 % CI 1.13-1.76, *P* < 0.01 for each 0.1 unit increase in ConI). This association remained significant when adjusted for demographics but not when fully-adjusted. Increasing ConI tertiles were also predictive of a greater risk of death in the crude model, although significance was lost following adjustment (Table 3).

### Gender Sub-analysis

When stratifying the data by gender (see Additional file 1: Table S1 and Table S2), a U-shaped association between BMI and the composite outcome was maintained for crude and multivariable analysis in both males and females. Model 2 and 3 lost statistical significance in the female group. The U-shaped association between BMI category and mortality was notably stronger in males over females, where the relationship was not significant at any level. For the composite outcome WC and ConI was not associated with the outcome for males or females in any model.

## Discussion

In the current study, BMI values in the overweight and class I/II obese range were shown to be associated with lower hazards of both the composite primary renal outcome and all-cause mortality in a population of Australian adults with stage 3–4 CKD. While a high ConI was predictive of the composite renal outcome in unadjusted models, neither WC nor ConI showed a significant association with kidney disease progression or mortality in adjusted models.

The outcome of this study, showing an inverse relationship between BMI and adverse outcomes, is similar to findings in previous studies investigating BMI and mortality in CKD patients [26–28]. An analysis of the Atherosclerosis Risk in Communities (ARIC) Study database demonstrated that a larger BMI was associated with better overall survival in a CKD population but an increased hazard of death in those without CKD [27]. Similarly, an evaluation of 12,534 individuals with stage 3–4 CKD participating in the Kidney Early Evaluation Program (KEEP) showed a survival advantage for patients who were obese, although the results were no longer significant when BMI exceeded 35 kg/m^2^ [28]. In contrast, the study showed no association between BMI and rates of progression to end-stage kidney disease. In patients with stage 3–4 CKD pooled from the Atherosclerosis Risk in Communities and Cardiovascular Health Study [29], obese BMI was protective against a composite outcome of cardiovascular events, stroke and all-cause mortality.

The association of overweight BMI with better clinical outcomes may be accounted for by the inability of BMI to discriminate body composition. An elevated BMI has the potential to represent better overall nutrition and high muscle mass, and an increased ability to adapt to the protein energy wasting state commonly observed in kidney disease. Moreover, higher BMI has been shown as protective in proteinuric CKD but not in non-proteinuric CKD [30]. BMI is also unable to account for differences in body mass distribution and the risks associated with increased visceral adiposity. For example, an analysis by Panwar and colleagues [31] using the Reasons for Geographic and Racial Differences in Stroke (REGARDS) Study data showed a higher BMI was associated with lower ESRD risk in those without, but not those with the metabolic syndrome. In this analysis, controlling for WC, which was more likely to reflect abdominal fatness, did not appreciably alter the relationship between BMI and the composite renal outcome and only slightly attenuated the association with mortality. Furthermore, the Tehran Lipid and Glucose Study (TLGS) [32], found that increases in weight, BMI, WC and hip circumference were associated with reduced mortality in men but not women. In a gender-stratified analysis of this data the U-shaped association between BMI and outcomes was found to be more pronounced in men than women but otherwise trends were maintained.

In contrast to the findings of the current study, Evans et al. [33] found that BMI was unrelated to time to RRT commencement in a Swedish population of 920 CKD patients, although the inverse relationship with mortality was maintained. Using data from the Modification of Diet in Renal Disease (MDRD) study [34], Madero et al. were also unable to find an independent association between BMI and mortality. However, in contrast to the population in the present study, the MDRD study excluded a number of important conditions that may have been impacted by the presence of obesity (including type 1 diabetes, insulin-dependent type 2 diabetes, autoimmune glomerulonephritis and renal artery stenosis) and potentially played a role in the different outcomes of the two studies.

Due to the limitations of BMI as a measure of obesity, it was hypothesised that more specific anthropometric measures of central obesity, including WC and ConI, may be better prognostic indicators than BMI. However, neither WC nor ConI were independent risk factors for kidney disease progression or death. This finding contrasts with that of a post-hoc analysis of 5,805 stage 1–4 CKD patients participating in the REGARDS Study, which showed that the highest WC group had an approximately two-fold increased hazard rate for all-cause mortality [19]. The apparent disparity in findings may be partly explained by the differences in the study populations. The REGARDS cohort was made up of more than 50 % individuals with stage 1–2 CKD, whilst the present study cohort was solely comprised of patients with stage 3 or 4 CKD. Participants categorised with very early CKD (eGFR ≥60 mL/min/1.73 m^2^) therefore may have been more closely matched to the risk profile of the general population than individuals with more advanced disease, explaining the deleterious effects of higher WC seen in their study. Furthermore, there is conflicting evidence surrounding the reliability of WC as a marker for visceral adiposity in CKD. Several studies have found different levels of correlation between visceral fat and measured WC.

In kidney disease especially, these inconsistent results may relate to the fluid disturbances which would alter the relationship between abdominal girth and visceral fat. Furthermore, there are methodological issues with the reproducibility of WC in the real world setting, more so than for BMI [35]. Panoulas and colleagues highlighted this problem in a study of the intra- and inter-operator variability of WC measurements, where there were significant differences between measurements by health practitioners [36]. While measurements in the current study were all recorded using a standardised protocol, there were a number of operators involved. The lack of significant correlation between WC and outcomes may indicate that the utility of WC is limited in real world settings.

ConI was also found to not be independently predictive of kidney disease progression or death in patients with stages 3–4 CKD. Although there have been no prior studies of ConI as a prognostic factor in non-dialysis CKD patients, Evans et al. reported that ConI was more strongly correlated with risk factors for cardiovascular disease and CKD progression, including eGFR, proteinuria, uric acid and systolic blood pressure, than WC or BMI [20]. A small study of 104 pre-dialysis CKD patients found that increasing ConI was associated with greater eGFR reduction over a 12 month period [21]. Furthermore, a study of 173 haemodialysis patients demonstrated an association between elevated ConI and mortality, which was no longer apparent following adjustment for markers of inflammation and malnutrition [18].

This study has a number of important limitations. Firstly, the observational design meant that a causal relationship could not be inferred from the observed associations. Secondly, the small sample size and limited study duration (median follow-up 3.23 years) meant that the possibility of a type 2 statistical error could not be excluded, particularly in the extreme BMI categories (<18.5 and ≥40 kg/m^2^). Nevertheless, 903 individuals participated in the study and 224 (25 %) experienced a primary event. Thirdly, there was limited adjustment for comorbidities in the models, such that the possibility of residual confounding could not be excluded. Fourthly, while there was a standard protocol used to measure waist circumference by trained health practitioners, the risk of inter-observer variation was not formally evaluated. Fifthly, the assessment of abdominal girth may have been confounded in patients with polycystic kidney disease, although such patients comprised less than 3 % of the total population. Sixthly, WC and ConI data were missing in approximately one-third of patients, which may have introduced bias. Finally, there were very few Aboriginal or Torres Strait Islander peoples within the study cohort, such that the results of the current investigation may not be generalisable to this high risk population [37].

## Conclusion

This study demonstrated that, in an Australian stage 3–4 CKD population, BMI in the overweight and obesity classes I/II range is associated with reduced risks of progression of renal disease and mortality, and that adjusting for WC does not significantly alter this association. Alternative anthropometric measures of central obesity (WC and ConI) were not significantly associated with disease progression or mortality in this population, however BMI may be valuable for risk-stratification of newly referred CKD patients.

## Abbreviations

ACR, Albumin:creatinine ratio; ANZDATA, Australia and New Zealand Dialysis and Transplant; BMI, Body mass index; CKD, Chronic kidney disease; CKD-EPI, Chronic Kidney Disease Epidemiology Collaboration; ConI, Conicity index; CVD, Cardiovascular disease; eGFR, Estimated glomerular filtration rate; HR, Hazard ratio; IQR, Interquartile range; MDRD, Modification of Diet in Renal Disease; PAH, Princess Alexandra Hospital; PCR, Protein:creatinine ratio; RRT, Renal replacement therapy; WC, Waist circumference

## Additional file

Additional file 1: Table S1. Association between baseline obesity parameters and the primary outcome in stage 3–4 chronic kidney disease stratified by gender. Comparison performed using Cox proportional hazards modelling to compare body mass index, waist circumference and conicity index with the composite outcome of doubling of serum creatinine, commencement of renal replacement therapy or all-cause mortality. **Table S2.** Association between baseline obesity parameters and all-cause mortality in stage 3–4 chronic kidney disease stratified by gender. Comparison performed using Cox proportional hazards modelling to compare body mass index, waist circumference and conicity index with all-cause mortality by gender. (DOCX 24 kb)
