# Supplementary material for: Association of anthropometric measures with kidney disease progression and mortality: a retrospective cohort study of pre-dialysis chronic kidney disease patients referred to a specialist renal service
Source: BMC Nephrol. 2016 Jul 8;17:74. doi: 10.1186/s12882-016-0290-y (PMC4939033; doi:10.1186/s12882-016-0290-y)
Supplement: Additional file 1: Table S1. — Association between baseline obesity parameters and the primary outcome in stage 3–4 chronic kidney disease stratified by gender. Comparison performed using Cox proportional hazards modelling to compare body mass index, waist circumference and conicity index with the composite outcome of doubling of serum creatinine, commencement of renal replacement therapy or all-cause mortality. Table S2. Association between baseline obesity parameters and all-cause mortality in stage 3–4 chronic kidney disease stratified by gender. Comparison performed using Cox proportional hazards modelling to compare body mass index, waist circumference and conicity index with all-cause mortality by gender. (DOCX 24 kb) [file 12882_2016_290_MOESM1_ESM.docx]

**Additional file 1**

**Table S1: Association between baseline obesity parameters and the primary outcome in stage 3-4 chronic kidney disease stratified by gender.** Comparison performed using Cox proportional hazards modelling to compare body mass index, waist circumference and conicity index with the composite outcome of doubling of serum creatinine, commencement of renal replacement therapy or all-cause mortality.

|  | **HR (95% CI)** | | | | | | | |
| --- | --- | --- | --- | --- | --- | --- | --- | --- |
|  | **Crude** | | **Model 1** | | **Model 2** | | **Model 3** | |
|  | **Female** | **Male** | **Female** | **Male** | **Female** | **Male** | **Female** | **Male** |
| **Body mass index (kg/m^2^)** | | | | | | | | |
| **<18.5** | 0.45 (0.1-1.9) | 1.30 (0.2-9.5) | 0.51 (0.1-2.2) | 1.51 (0.2-11.1) | 0.31 (0.04-2.4) | 1.64 (0.2-12.1) | 0.40 (0.05-3.3) | 2.65 (0.4-20.2) |
| **18.5-24.9** | 1.00 (referent) | 1.00 (referent) | 1.00 (referent) | 1.00 (referent) | 1.00 (referent) | 1.00 (referent) | 1.00 (referent) | 1.00 (referent) |
| **25-29.9** | 0.54 (0.3-1.0)* | 0.57 (0.4-0.9)* | 0.54 (0.3-1.0)* | 0.55 (0.4-0.9)* | 0.6 (0.3-1.1) | 0.58 (0.4-0.9)* | 0.52 (0.3-1.1) | 0.48 (0.3-0.8)* |
| **30-39.9** | 0.65 (0.4 -1.2) | 0.62 (0.4-1.0)* | 0.67 (0.4-1.2) | 0.64 (0.4-1.0) | 0.8 (0.4-1.5) | 0.66 (0.4-1.1) | 0.59 (0.3-1.2) | 0.63 (0.4-1.1) |
| **≥40** | 0.51 (0.2-1.3) | 0.96 (0.5 -1.8) | 0.55 (0.2-1.4) | 1.11 (0.6-2.1) | 0.56 (0.2-1.5) | 1.29 (0.7-2.5) | 0.54 (0.2-1.6) | 1.32 (0.6-2.7) |
| **Waist circumference tertiles (cm)** | | | | | | | | |
| **M: <98.5, F<90** | 1.00 (referent) | 1.00 (referent) | 1.00 (referent) | 1.00 (referent) | 1.00 (referent) | 1.00 (referent) | 1.00 (referent) | 1.00 (referent) |
| **M: 98.5-110,**  **F: 90-101** | 1.06 (0.6-2.0) | 1.36 (0.8-2.3) | 1.02 (0.5-1.9) | 1.17 (0.7-1.9) | 1.2 (0.6-2.3) | 1.37 (0.8-2.3) | 1.02 (0.5-2.2) | 0.99 (0.65-1.8) |
| **M>110, F: >101** | 0.81 (0.4-1.6) | 0.91 (0.5-1.6) | 0.84 (0.4-1.7) | 0.9 (0.5-1.5) | 0.81 (0.4-1.8) | 1.12 (0.6-2.0) | 0.8 (0.3-1.9) | 0.95 (0.5-1.7) |
| **Conicity index tertiles** | | | | | | | | |
| **<1.291** | 1.00 (referent) | 1.00 (referent) | 1.00 (referent) | 1.00 (referent) | 1.00 (referent) | 1.00 (referent) | 1.00 (referent) | 1.00 (referent) |
| **1.291-1.372** | 1.05 (0.6-2.0) | 1.24 (0.7-2.3) | 1 (0.5-1.9) | 0.93 (0.5-1.8) | 1.07 (0.6-2.1) | 1.06 (0.5-2.1) | 1.23 (0.6-2.5) | 1.04 (0.5-2.2) |
| **>1.372** | 1.56 (0.8-3.1) | 1.66 (0.9-3.0) | 1.48 (0.8-2.9) | 1.28 (0.7-2.4) | 1.15 (0.5-2.5) | 1.61 (0.8-3.1) | 1.37 (0.6-3.3) | 1.09 (0.5-2.2) |

Results expressed as hazard ratio (95% confidence interval).

Model 1: Adjusted for age. Model 2: Adjusted for age, gender, race (Caucasian vs. Non-Caucasian). Model 3: Model 2 + estimated glomerular filtration rate, proteinuria, cause of chronic kidney disease, diabetes status.

* P ≤ 0.05, ** P ≤ 0.01

**Table S2: Association between baseline obesity parameters and all-cause mortality in stage 3-4 chronic kidney disease stratified by gender.** Comparison performed using Cox proportional hazards modelling to compare body mass index, waist circumference and conicity index with all-cause mortality by gender.

|  | **HR (95% CI)** | | | | | | | |
| --- | --- | --- | --- | --- | --- | --- | --- | --- |
|  | **Crude** | | **Model 1** | | **Model 2** | | **Model 3** | |
|  | **Female** | **Male** | **Female** | **Male** | **Female** | **Male** | **Female** | **Male** |
| **Body mass index (kg/m^2^)** | | | | | | | | |
| **<18.5^†^** | - | 1.93 (0.3-14.3) | - | 1.79 (0.2-13.3) | - | 1.87 (0.3-14.0) | - | 2.62 (0.3-20.4) |
| **18.5-24.9** | 1.00 (referent) | 1.00 (referent) | 1.00 (referent) | 1.00 (referent) | 1.00 (referent) | 1.00 (referent) | 1.00 (referent) | 1.00 (referent) |
| **25-29.9** | 0.61 (0.31-1.20) | 0.53 (0.3-0.9)* | 0.59 (0.3-1.2) | 0.51 (0.3-0.9)* | 0.66 (0.3-1.4) | 0.53 (0.3-0.9)* | 0.62 (0.3-1.5) | 0.45 (0.2-0.9)* |
| **30-39.9** | 0.73 (0.4-1.4) | 0.41 (0.2-0.7)* | 0.82 (0.4-1.6) | 0.50 (0.3-0.9)* | 1.08 (0.5-2.2) | 0.49 (0.3-0.9)* | 1 (0.4-2.3) | 0.49 (0.2-0.1)* |
| **≥40** | 0.56 (0.2-1.5) | 1.16 (0.6-2.4) | 0.78 (0.3-2.2) | 2.07 (0.9-4.4) | 1.02 (0.4-3.0) | 2.4 (1.1-5.1)* | 1.4 (0.5-4.4) | 1.84 (0.8-4.3) |
| **Waist circumference tertiles (cm)** | | | | | | | | |
| **M: <98.5, F<90** | 1.00 (referent) | 1.00 (referent) | 1.00 (referent) | 1.00 (referent) | 1.00 (referent) | 1.00 (referent) | 1.00 (referent) | 1.00 (referent) |
| **M: 98.5-110,**  **F: 90-101** | 1.27 (0.6-2.6) | 1.15 (0.6-2.2) | 1.18 (0.6-2.4) | 0.93 (0.5-1.8) | 1.32 (0.6-2.8) | 1.06 (0.5-2.1) | 1.16 (0.5-2.8) | 0.68 (0.3-1.5) |
| **M>110, F: >101** | 0.92 (0.4-2.0) | 0.78 (0.4-1.6) | 1.12 (0.5-2.5) | 0.91 (0.5-1.8) | 1.12 (0.5-2.7) | 1.02 (0.5-2.1) | 1.4 (0.5-3.8) | 0.72 (0.3-1.7) |
| **Conicity index tertiles** | | | | | | | | |
| **<1.291** | 1.00 (referent) | 1.00 (referent) | 1.00 (referent) | 1.00 (referent) | 1.00 (referent) | 1.00 (referent) | 1.00 (referent) | 1.00 (referent) |
| **1.291-1.372** | 1.2 (0.6-2.4) | 4.4 (1.3-14.7)* | 1.05 (0.5-2.1) | 2.3 (0.7-7.8) | 1.08 (0.5-2.2) | 2.27 (0.7-7.7) | 1.32 (0.6-2.9) | 2.32 (0.7-8.3) |
| **>1.372** | 1.78 (0.8-3.8) | 4.63 (1.4-15.3)* | 1.56 (0.7-3.3) | 2.59 (0.8-8.6) | 1.19 (0.5-2.8) | 2.61 (0.8-8.8) | 1.42 (0.6-3.7) | 1.59 (0.4-5.8) |

Results expressed as hazard ratio (95% confidence interval).

Model 1: Adjusted for age. Model 2: Adjusted for age, gender, race (Caucasian vs. Non-Caucasian). Model 3: Model 2 + estimated glomerular filtration rate, proteinuria, cause of chronic kidney disease, diabetes status.

* P ≤ 0.05, ** P ≤ 0.01

†No HR available for female BMI category <18.5 as no death recorded.
